# Use of a Portable Inertial Measurement Unit as an Evaluation Method for Supraspinatus Muscle: Proposed Normative Values

**DOI:** 10.3390/s21227723

**Published:** 2021-11-20

**Authors:** Sagrario Pérez-de la Cruz

**Affiliations:** Department of Nursing, Physical Therapy and Medicine, University of Almería, La Cañada de San Urbano, 04120 Almería, Spain; spd205@ual.es; Tel.: +34-950-21-45-74

**Keywords:** rehabilitation, range of motion, shoulder

## Abstract

Treatment protocols do not specify an appropriate weight for rehabilitating the shoulder joint. The purpose of this study was to establish normative values for the shoulder abduction range of motion and recommended weights to be used in the rehabilitation process after injury to the supraspinatus muscle. Fifty-eight volunteers were assessed using the DyCare system. A test was conducted by lifting the arm to a 90° angle and having the participants lift different weights. The range of motion was similar for both sexes, suggesting that sex had no influence on this variable. Regarding the use of weights, men did not show as much stability in their movement execution, with a high dispersion seen in values between zero and three kilograms of weight, reaching a maximum weight of six kilograms. However, women showed good joint stability from the beginning of the test, with values that remained constant as weight increased up to a maximum of five kilograms. In conclusion, no major differences were observed in supraspinatus muscle injury recovery according to sex. However, differences were observed in the amount of weight that was necessary and appropriate to allow the participants to recover their muscular strength and avoid relapses.

## 1. Introduction

Of all injuries that are commonly reported in clinical practice, tendon injuries involving the supraspinatus muscle are one of the most frequent causes of pain and functional impairment in the shoulder among young adults [1,2,3,4,5,6]. The supraspinatus muscle is commonly injured in sports requiring the use of the upper limbs due to excessive movement and the lack of stability inherent to its structure [5]. In primary care in Spain, this condition affects one fifth of the population, reaching an incidence of between approximately 11% and 39% of young adults who practice some type of physical activity and/or sports [2]. These injuries have an important impact on healthcare and lead to increased costs in the health system. Restoring shoulder function after surgical or conservative treatment is essential for patients and the preservation of functional independence. Recovering optimal levels of strength is crucial in common clinical practice [7]. Research on measuring strength and range of motion (ROM) is necessary in order to improve the recovery from and prevention of injuries caused by overuse, as well as to stabilise movement throughout the functional and extreme articular range and after a period of immobilisation following surgical intervention or rest.

Carrying out the right exercises with suitable progress, intensity, frequency, and loads will achieve to a decrease in relapse injuries during recovery, especially overuse injuries. Therefore, pinpointing risk factors is key to setting up preventative, health promotion, and recovery programmes [1]. Studies are currently advocating active exercise to treat tendinopathy [8,9], which includes strengthening joint stabilisers [10] and providing neuromuscular training for the shoulder complex [11,12,13,14,15,16,17]. No consensus about the most suitable exercise strategy (recovery of strength with the incorporation of weights) or standardised protocol for this type of injury has been reached to date. Previous research has suggested that normative ROM data are needed for individuals engaging in any physical activity because little data are available on this topic [18,19]. More research will be needed to develop evidence-based exercise programmes and indicate weights and speeds that can be recommended and applied to patients. In addition, while many more studies on shoulder injuries and ranges of motion in sport are needed, even fewer studies have focused on clinical practice. 

The objective of this study was to establish normative values for abduction shoulder ROM and recommend weights to be used in the rehabilitation process after injury to the supraspinatus muscle. The shoulder joint was selected for this initial investigation because its use is required for participation in many common sports. Furthermore, this study sought to analyse the possible influential anthropometric factors that would define predictive values for young adults.

## 2. Materials and Methods

### 2.1. Participants

In total, 76 young adult students from Spain’s National Police School, ENP (Escuela de Nacional de Policía), and the University of Almeria’s Faculty of Health Sciences volunteered to take part in this study. Each participant was asked to sign an informed consent form before the study. Ethical approval for this cross-sectional study was granted by the Bioethics Committee of the University of Almeria (Ref: UALBIO2020/022) following authorisation from the ENP director, the ENP’s Department of Physical Education director, and the University of Almeria. The study was conducted between October 2019 and March 2020. The inclusion criteria were as follows: individuals aged 18 and older; ability to work with the verbal indications and motor actions requested during the test; and no prior history of surgery, trauma, or pain in the left or right shoulder joint in the last six months. The following exclusion criteria were applied: worsening of a general state of health or condition, articular limitation in the upper limb (shoulder, elbow, hand), and pain during the test. Of the 76 volunteers, 58 were included in the final sample. 

### 2.2. Measuring Instruments

Inertial measurement units (IMUs) were used for measuring the range of motion in the shoulder joint (DyCare^®®^ Lynx, Barcelona, Spain). The IMUs had two sensors, each with a gyroscope, accelerometer, and magnetometer, and worked at a sampling frequency of 102.4 Hz. The sensor dimensions were 50 mm × 34 mm × 14 mm and the sensors were configured as follows: the gyroscope at 2000°/s, the accelerometer at 2 g, and the magnetometer at 4.7 Ga. The data were collected and the IMU system was processed using the DyCare^®®^ Lynx software (version 1.7.0) provided by the manufacturer. To calculate the joint angle, the emitted signals were transformed into quaternions—a four-part vector that stores a rotation—using a fusion algorithm based on work by Madgwick. Experiments were recorded using the “free joint” mode. 

### 2.3. Procedure

The procedure was explained to the study participants in detail and questions related to the study were answered before the measurements were taken. Participants were informed that they had the right to interrupt the procedure at any time upon request. The test lasted approximately 20 min. Weight, height, and body mass index were recorded for each individual, as were laterality (by the Harris test) and hours of physical activity per week.

**Figure 1:**
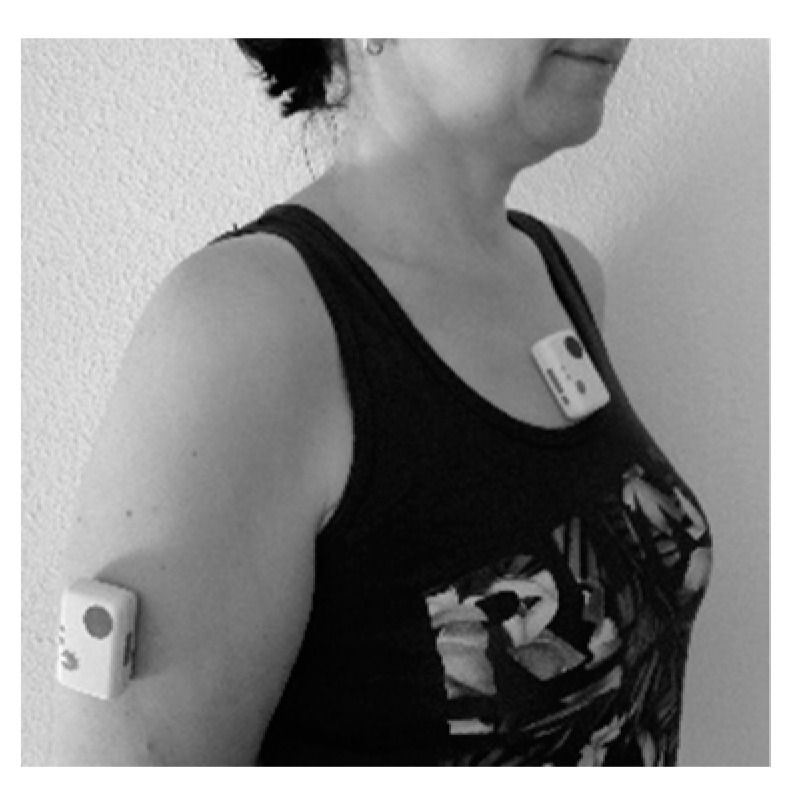
Sensor placement.

Both measurement systems (sensors) were applied to the skin, on the middle third of the arm and trunk at the sternum, with double-sided hypoallergenic adhesive tape (Figure 1). Care was taken to guarantee that the sensors were well placed following the system’s protocol (with a distance between both devices of no less than 3 cm). The subject’s body was also stabilised to minimise compensations in other parts of the body that could modify the results obtained in the measurement.

The individual’s shoulder joint was uncovered to avoid clothing interfering with signals. The participants were permitted three submaximal tests prior to data collection in order to familiarise themselves with the testing equipment and procedures. The test protocol consisted of lifting the arm (abduction movement) with five repetitions at an angular velocity of 60°/s. This was followed by a two-minute resting period between each test. Each series was assessed with progressive loads to evaluate ROM and velocity with varying loads (to recover strength from an injury, progressive loads are used depending on the individual’s tolerance level). This protocol has been described in previous studies, such as research by Samah Mamoud in children [20]. The test was ended if volunteers reported a pain level of 5 or higher on the pain scale.

### 2.4. Statistical Analysis

The data were analysed with the Statistical Package for Social Sciences (SPSS, version 25) software for Windows and the Visual Studio Code in the Python language. A Kolmogorov–Smirnov test with a 95% confidence interval (CI) was carried out to establish the normality of continuous data distributions. The data were analysed by the use of an unpaired t-test to assess differences between sexes, the Pearson correlation coefficient to assess correlation, and the analysis of variance to assess differences between genders. Differences were considered statistically significant at *p* < 0.05. Descriptive statistics, including the mean, standard deviation, and outliers, were calculated for each study variable. 

## 3. Results

The final sample was composed of 58 patients, of whom 72.41% were men (*n* = 42) and 27.58% were women (*n* = 17) aged between 23 and 38 years old. The mean age was 23.23 (SD = 3.5), and 52 individuals were right-handed (89.65%) and 6 were left-handed (10.34%). Table 1 presents the anthropometric characteristics of each individual.

**Figure 2:**
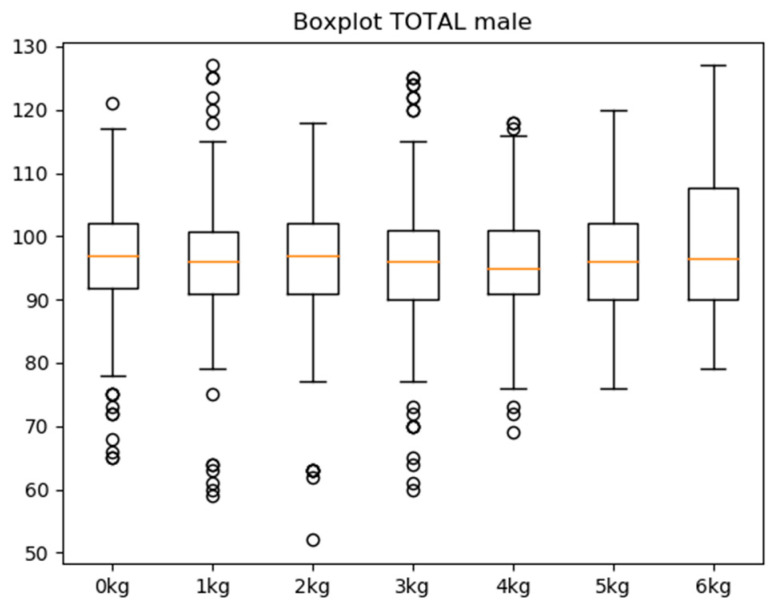
Variability of joint width by weight (male).

**Figure 3:**
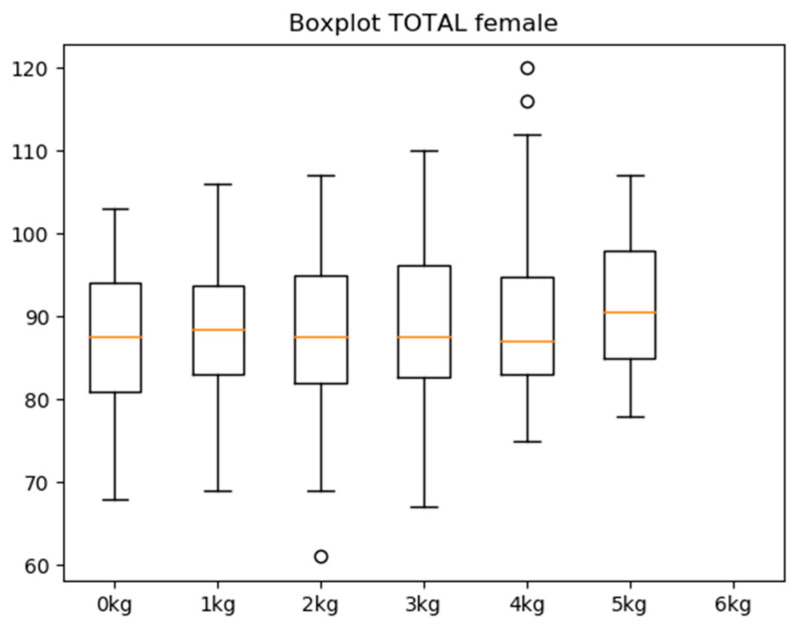
Variability of joint width by weight (female).

Among participants who were able to lift every weight, the ROM (the dependent variable) by weight was very similar. Among men, the average median ROM of each weight was 96.21 degrees (SD ± 0.76), whereas the average among women was 88.08 degrees (SD ± 1.28) (Table 2). The Kolmogorov–Smirnov test confirmed the normality of the ROM (Z = 1.009; *p* > 0.2). Men reached 8.13 degrees more than women. The difference between weights with the highest and lowest ROM among women was 4.5 degrees for the mean and 3 degrees for the median. In men, the difference was 3.14 degrees for the mean and 2 degrees for the median (Figure 2 and Figure 3). There was no correlation (r = 0.19; *p* = 0.04) between age and abduction. No other relationship or difference due to age, gender, or interactions reached significance. Sex differences in the range of motion were analysed, showing significant differences in relation to the amount of weight they could move, which was higher in the case of men (r = 0.35; *p* < 0.05) (Table 3).

It cannot be said, however, that there were differences between weights, as the degree range may be produced by sensor system variability and trembling in individuals, among other factors.

**Figure 4:**
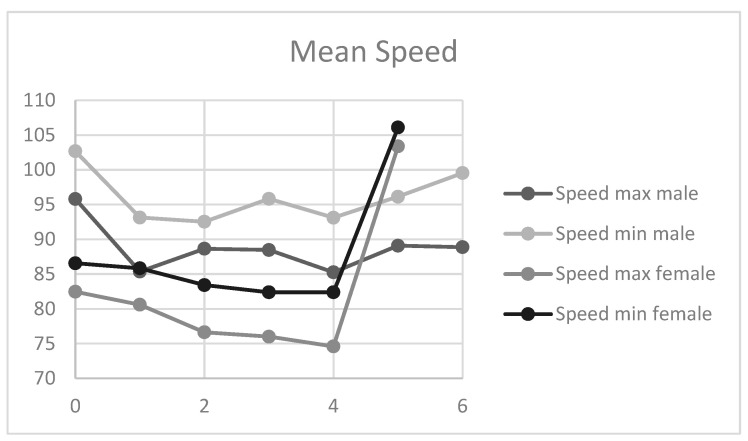
Mean speed of execution of movement; *y*-axis: m/s; *x*-axis: kg.

In relation to velocity, the mean maximum and minimum velocity did not show any clear trend among men or women and there was very little variation, except for the maximum of 5 kg in women (Figure 4).

**Figure 5:**
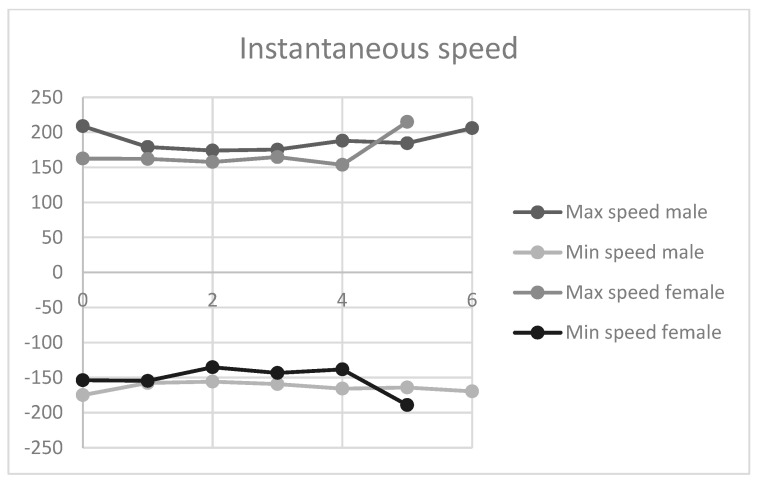
Instantaneous speed of execution of movement; *y*-axis: m/s. Shoulder abduction movement; *y*-axis: kg.

Another important point to note is the volunteers’ maximum weight lifted during the test (a dependent variable). In men, the maximum was 7 kg, with reduced stability in movements with a load between 0 and 3 kg. They reached a maximum of 7 kg before showing any signs of danger of a supraspinatus muscle injury relapse (pain when carrying out tasks and inability to achieve full joint range). Women, however, showed a good joint stability from the start of the test, and this remained constant as weight increased up to a maximum of 6 kg (*p* < 0.05) (Figure 5).

## 4. Discussion

Among the working population, work involving the movement of the shoulder above 90° is linked to a greater risk of tendinopathy in the rotator cuff [21,22]. Evidence presented in biomechanical studies supports these findings, showing that the intramuscular pressure in rotator cuff muscles increases when the arm is lifted excessively [23]. According to the Dutch protocol guide for the correct diagnosis and treatment of subacromial pain syndrome, therapy with exercise is more effective than any other treatment to reduce pain and improve shoulder function [24]. Exercises specifically geared to the rotator cuff and scapular stabilisers appear to be more effective than general exercise therapy [12]. 

One of the essential components of rehabilitation programmes for patients with shoulder impingement syndrome is therapeutic exercise, which is now considered to be an effective intervention for this condition [25,26,27,28,29,30,31,32,33,34,35]. Despite the limited evidence of the effectiveness of physical means, the results suggest that, rather than the physical means used, the most important factor in functional recovery in patients with shoulder impingement syndrome is exercise [28]. For instance, Kooijman et al. (2013) [29] studied the effectiveness of physical therapy in patients with this shoulder condition and found clinical improvement in 64% of patients [30]. As a result, emphasis should be placed on therapeutic exercise at the earliest stages of care for patients with shoulder impingement syndrome, as it provides a greater chance of improvement and fewer complications.

Progressive heavy-slow resistance exercise programmes have been recommended due to their similarity to daily activities, which presumably increases the likelihood of compliance. Eccentric rotator cuff muscle exercises can induce a less suitable initial position, reducing subacromial space and increasing the anticipated risk of tendon clamping [36,37].

Alternatively, using exercises that start with a concentric phase and only allowing patients to perform isometric exercises when limited by pain could potentially minimise the risk of irritating structures within the subacromial space [38]. This study determined the mean range of motion for shoulder abduction, which is the main activity needed for the upper limb to function, in men and women. No statistically significant differences by sex are reported. In a recent report that used an optical motion capture system to examine daily work in the upper limb, data provided for men and women were highly consistent with our findings—there was a difference of 0.4° in ROM [39]—and with results obtained in studies by Safaee-Rad et al. [35] and Kouchi et al. [36], with differences below 5°. However, the maximum abduction of the shoulder joint, internal rotation, forearm pronation, and wrist extension angles and ROM of shoulder joint flexion and abduction, elbow joint flexion, and radial flexion of the wrist joint were not similar to results reported by Safaee-Rad et al. [35], with differences of over 5°. We believe that these differences are due to the use of different measurement tools and differences in individuals’ posture between this previous study and our own. In this study, the ROM between men and women was the same, although a difference was found in the weight they were able to lift. This difference of 8° between men and women is not high enough to state that sex should be a determining factor in putting together a shoulder rehabilitation programme (for restoring the supraspinatus muscle). The work protocol may be the same for both sexes but dependent on the weight used in recovery.

After defining the work protocol for restoring movement in the glenohumeral joint in supraspinatus muscle lesions, it is important to determine the value of the weights that need to be lifted to recover muscular strength.

A strong recommendation can be made to prescribe exercise to patients affected by supraspinatus injury. However, the most suitable exercise regimen remains unclear, as many clinical trials and systematic reviews do not describe such exercise programmes in detail. For example, whether treatment should be designed around loads that may temporarily reproduce and aggravate patients’ pain and symptoms remains a subject of debate [40]. Most of the researched forms of physiotherapy-led interventions have been applied as standard protocols without taking into account individual needs and may therefore have limited effect. These interventions are informed by surveys of instructions given by physical therapists for rehabilitating musculoskeletal shoulder problems. The following principles are the most used [41]: exercises can be performed at home and/or at a clinic; patients may experience some discomfort (below 5/10 on a visual analogue scale); exercises should include resistance; and 12 weeks is the expected duration of therapy. Further research into the prescription of different types of exercises for managing these injuries is required to provide clear instructions and recommendations. Future reviews and research should focus on exercise therapy (e.g., types, number of repetitions, etc.).

According to the proposal by the rehabilitation council in the Netherlands [24], using weights in exercises is more effective than any other treatment to reduce pain and improve shoulder function [25,37]. Exercises specifically geared towards the rotator cuff and shoulder girdle stabiliser muscles appear to be more effective than general upper limb exercise programmes [39]. Also described is the lack of difference in the effectiveness of attending physiotherapy sessions as an outpatient and at-home therapy [42,43].

High-load progressive exercise programme has been optimised in many ways that set it apart from traditional eccentric exercise programmes. Concentric heavy-slow resistance exercise programmes are recommended due to their similarity to daily activities, which presumably increases the likelihood of compliance. Progressive high-load exercises have been found to offer a significant benefit as opposed to low-load shoulder exercises [33]. A set of repetitions with progressive loads, as employed in this study, can help doctors to treat patients diagnosed with tendinopathy in the rotator cuff while emphasising static and stability work in the shoulder joint complex to avoid injury relapse. According to the results obtained in our study, male patients showed less stability in executing the arm separation, with a large dispersion seen in values between 0 and 3 kg of weight, but with improved joint stability and velocity from 4 kg to the maximum weight that they were able to lift. However, women showed good motor action execution and joint stability from the start of the test, and these remained constant throughout the test as the weight increased. None of the individuals were able to lift over 7 kg, while women were not able to lift amounts exceeding 6 kg. The standard deviation in the sample was very small, indicating that the weight increase did not result in a higher ROM or greater strength in execution. Throughout the test, the ROM or greater strength remained constant. 

Recovering shoulder ROM, restoring muscular strength, and enabling the use of the arm in daily life again are the objectives of postoperative treatment and rehabilitation programmes.

Currently, other appropriate therapeutic actions, together with mobilisations, include the use of focal vibration as an element that induces an increase in excitability during muscle contraction, which leads to a better and greater motor response. It will be interesting to incorporate a focal vibration for a few minutes prior to the motor action into the protocols for this pathology [44]. However, until now, few studies have advised or provided guidance on quantitative progressive workload values used for patients recovering from supraspinatus muscle injuries [39,45]. The results obtained in this article may be considered suitable benchmarks for rehabilitation professionals in their daily work. Considering the results of this study, we could extrapolate the findings found in the healthy population to propose protocols in patients with similar characteristics. This proposal may be extrapolated to collective work environments, since offering group sessions with patients would reduce healthcare costs while providing efficient, prompt care for this common condition, with no loss of specificity or validity.

Regarding the limitations of this study, it would be advisable to expand the sample to include patients with a wider age range and with other previously existing conditions to confirm that the information presented in this study can be extrapolated to a wider population.

## 5. Conclusions

This study identifies some significant sex-based characteristics in relation to ROM and weight capacity in healthy young adults. The functional joint range hardly varies in relation to the sex of the sample. Conversely, regarding the amount of weight that could be moved, the amount men could lift did not exceed six kilograms, whereas the amount women could lift did not exceed five kilograms, revealing specific values by sex for rehabilitating the strength component in supraspinatus tendon injuries. According to the results of this study, a relationship between anthropometric factors with predictive values cannot be presumed. This research provides some benchmarks that may serve as guidelines for professional decision making in clinical practice.

## Figures and Tables

**Table 1:** Anthropometric variables in the sample.

	Min–Max	Mean ± SD
Age	23–38	28.32 ± 3.5
Height	162–194	175.91 ± 7.11
Weight	53–92	73.81 ± 8.81
Physical activity (hours/week)	4–18	7.60 ± 2.40
BMI	18.7–29.1	23.65 ± 3.59

BMI: body mass index; SD: standard deviation.

**Table 2:** Evaluation of range of motion (ROM) with different weights by gender.

Sex	Mean (Degrees °)	SD
Female	88.08	1.28
Male	96.21	0.76

**Table 3:** Evaluation of mean lifting and lowering speeds for different weights by gender. Instantaneous velocities exhibited a stable trend, with little variation seen between weights.

Sex	Mean Lifting Speed (°/s)	SD	Mean Lowering Speed (°/s)	SD
Female	78.62	10.75	84.63	9.14
Male	88.76	3.84	95.83	3.77
